# Fluorescence ‘Turn-on’ Probe for Chromium Reduction, Adsorption and Detection Based on Cellulosic Nitrogen-Doped Carbon Quantum Dots Hydrogels

**DOI:** 10.3390/gels10050296

**Published:** 2024-04-25

**Authors:** Hebat-Allah S. Tohamy

**Affiliations:** Cellulose & Paper Department, National Research Centre, 33 El-Bohouth St., Dokki, Giza P.O. 12622, Egypt; hs.tohamy@nrc.sci.eg or hebasarhan89@yahoo.com

**Keywords:** fluorescence ‘turn-on’ probe, Cr(VI) reduction, liquid microwave method, adsorption, chemosensors, limit of detection

## Abstract

This paper proposes a new, highly effective fluorescence test for Cr(VI) detection. This method utilizes a hydrogel composed of hydroxyethyl cellulose (HEC), nitrogen-doped carbon quantum dots (N–CQDs), and poly(co-acrylamido-2-methyl-1-propane sulfonic acid) (AMPS). The N–CQDs were successfully synthesized using a simple microwave method, and then conjugated with HEC and AMPS. The higher adsorption (99.41%) and higher reduction rate in H1 likely stems from both the presence of N–CQDs (absent in HB) and their increased free functional groups (compared to H2/H3, where N–CQDs block them). This facilitates the release (desorption) of Cr(VI) from the hydrogels, making it more available for reduction to the less toxic Cr(III). The fluorescent brightness of the HEC-N–CQDs-g-poly(AMPS) hydrogel increases gradually when Cr(VI) is added in amounts ranging from 15 to 120 mg/L. The fluorescent enhancement of the HEC-N–CQDs-g-poly(AMPS) hydrogel appeared to exhibit a good linear relationship with the 15–120 mg of the Cr(VI) concentration, with a detection limit of 0.0053 mg/L, which is lower than the standard value published by WHO. Our study found that the HEC-N–CQDs-g-poly(AMPS) hydrogel served effectively as a fluorescent probe for Cr(VI) detection in aqueous solutions, demonstrating high sensitivity.

## 1. Introduction

Water treatment and sensing underpin our connected world, driving innovation and improving lives [1,2]. Though essential for healthy ecosystems, hexavalent chromium (Cr(VI)), widely used in industry, poses a serious threat due to its extreme toxicity [2,3]. In order to address this environmental and health concern, converting this harmful form to the less toxic trivalent chromium (Cr(III)) through a reduction process is the most effective solution [4]. Scientists have been widely studying metal nanoparticles (M-NPs), particularly gold and silver, because of their unique properties. Unfortunately, conventional methods for preparing M-NPs, like high-energy ball milling and ultrasonic shot peening, have their drawbacks. These techniques can be costly, take a long time, involve hazardous materials, and harm the environment [5]. Carbon quantum dots (CQDs) could be used for reducing Cr(VI) safely [6]. It was found that nitrogen-doped carbon quantum dots (N–CQDs) emit a stronger fluorescence compared to previously reported fluorescent sensors based on CQDs [3,7]. For this reason, we will recycle N–CQDs from agricultural waste as an eco-friendly reducing agent. N–CQDs are spherical nanodots that combine diamond-like sp3 carbon and flat sheets of sp2 carbon. They can be prepared using an eco-friendly microwave method on agricultural waste [2,8,9,10,11,12]. N–CQDs have attracted significant attention in chemosensing and adsorption applications due to their unique properties, including their minute size, optical properties, excellent hydrophilicity, tailorable surface chemistry, cost-effective production, and straightforward fabrication. The various active functional groups on their surface make them excellent candidates for adsorbing different metal ions [2]. It was found that N–CQDs have improved fluorescence emission compared to CQDs [2,13]. Adsorbents with sensing capabilities in all forms empower water purification and environmental health [2]. Sensing applications have transformed the way we interact with our surroundings [1]. Sensing technologies, such as chemosensors for metal ions and sophisticated adsorbents, give us valuable information about our surroundings, allowing us to manage resources more efficiently, enhance safety protocols, and provide superior healthcare solutions [2,14]. Advances in combining adsorption and sensing technologies are not just driving industrial progress, but are also key to creating sustainable solutions for environmental issues like pollution and resource scarcity [2]. Separating N–CQDs after use can be tricky. In order to address this, we explored incorporating them directly within the hydrogel, simplifying the separation process. With technological progress, there is a growing need for sustainable alternatives in material science. Cellulose, a naturally abundant and eco-friendly resource, is a key area of research for creating the next generation of materials. Recently, scientists and businesses have prioritized finding sustainable and environmentally friendly materials. They are increasingly seeking innovative materials that combine cutting-edge performance with ecological awareness [15,16]. As the global need for water treatment and sensing technologies grows, so does the need for materials that are both high-performing and sustainable [2]. Cellulose-based hydrogels have recently emerged as a promising class of materials for adsorption and sensing applications, due to their unique properties and potential to meet the growing demand for sustainable and high-performing materials in these fields [8]. Cellulose, a complex sugar molecule that naturally forms lengthy chains, is one of the most common organic molecules found on Earth. It is derived from renewable resources, making it a sustainable and versatile material [17,18,19]. Cellulose molecules have both water loving and water hating regions. The hydrophilic groups are located in the equatorial positions of the glucopyranose ring, while the hydrophobic groups are located in the axial positions [17]. Hydroxyethyl cellulose (HEC) is a safe, non-reactive, biocompatible, tasteless, and water-soluble cellulose derivative [8,18]. Sustainable fluorescent N–CQDs prepared from mature beech pinewood sawdust (MB) are commonly used to make HEC-based hydrogels. Hydrogels are water-swelling networks of polymers that can absorb water without dissolving [8]. Many researchers have recently explored chemical, physical, and biological methods to reduce the toxicity of wastewater. These methods include biological treatments, electrocoagulation, aerobic biological treatments, and photocatalysis. Of these methods, adsorption is the most effective for removing heavy metals, as it does not require high temperatures, specialized equipment, or significant energy input [2]. 

Tohamy et al. demonstrated that carbon quantum dots (CQDs) can remove 83.85% of Cr(VI) from water [2]. Furthermore, the hydrogel produced when N–CQDs are crosslinked with HEC and 2-acrylamido-2-methyl-1–propane-sulfonic acid (AMPS) (also known as HEC–N–CQDs–g–poly(co–AMPS)) in our previous published work investigated how the dynamic viscoelastic properties of hydrogel changed after N–CQDs were incorporated into the hydrogel. Over a broad range of angular frequency area, both moduli exhibited significant change, and the nature of HEC-g-poly(co-AMPS) (i.e., HB without N–CQDs) changed from elastic to viscous. On the other hand, the cross-linking and bonding contacts within the HEC-N–CQDs–g-poly(co-AMPS) with a high N–CQD content become stronger, indicating that the elastic property predominates over the viscous characteristic. A HEC hydrogel with stable fluorescence properties was produced as a result of our previously published work, the evenly distributed dispersion of the fluorescent N–CQDs in the hydrogel, and the strong electrostatic interaction between the –NH_2_ functional group of N–CQDs and the –COOH of HEC, which help in the hydrogel networking formation, thereby maintaining the hydrogel’s integrity while preserving the N–CQDs’ fluorescence properties. The hydrogel that was produced by crosslinking N–CQDs with HEC and AMPS has excellent properties such as low toxicity, good biocompatibility, and low cost, as it did not require specialized equipment [8]. Investigating their application in the domains of Cr(VI) reduction to Cr(III), adsorption, and fluorescence ‘turn-on’ probes for Cr(VI) can broaden the range of uses for the HEC-N–CQDs-g-poly(AMPS).

## 2. Results and Discussion

**Figure 1:**
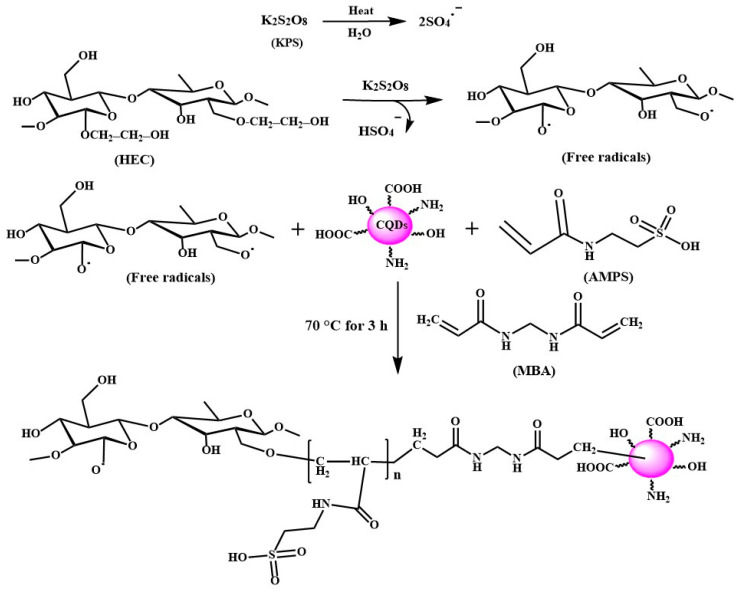
Reaction mechanism for synthesizing hydroxyethyl cellulose-N–CQDs-g-poly(co-AMPS) hydrogels.

Grafting CQDs and AMPS onto HEC in the presence of KPS as a free radical initiator and MBA as a crosslinking agent resulted in the creation of a hydrogel. The hydrogel absorbed 1372.18, 1862.43, and 1627.54% for H1, H2, and H3, respectively, which was 177.75, 241.25 and 210.83% more than its blank hydrogel without N–CQDs (i.e., H1; 771.96%), respectively [1]. Figure 1 details a step-by-step explanation of how the grafting and chemical crosslinking reactions are believed to occur. When heated, the KPS initiator breaks down into sulfate anion radicals (SO4^•−^). Alkoxy radicals are created on HEC by the SO4^•−^ radicals taking hydrogen away from the hydroxyl group. AMPS monomer molecules near the reaction sites accept HEC radicals, initiating chains. These chains donate free radicals to nearby N–CQD molecules, enabling the grafted chain to propagate [20,21]. MBA is a crosslinking agent, so its end vinyl groups may react with polymer chains during chain propagation. This creates a crosslinked copolymer [20]. 

### 2.1. Adsorption Study

**Figure 2:**
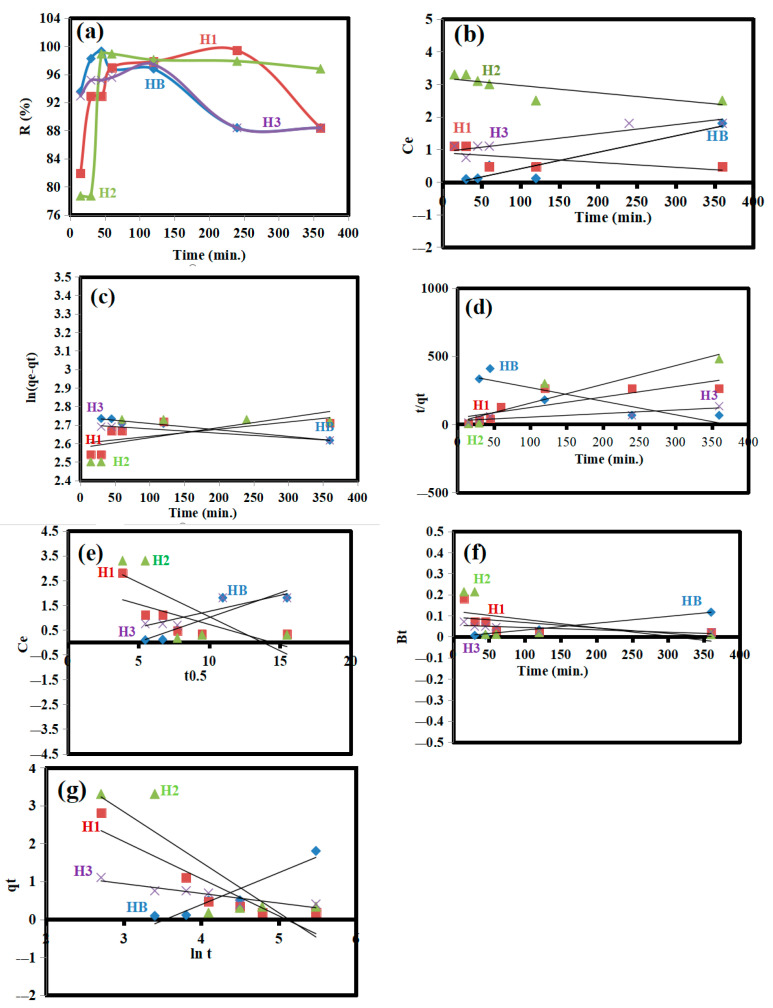
(**a**) Effect of contact time, (**b**) zero-order reaction, (**c**) pseudo-first-order, (**d**) the pseudo-second-order, (**e**) Weber–Morris, (**f**) Boyd, and (**g**) Elovich models on HB, H1, H2, and H3.

Hydrogel samples HB, H1, H2, and H3 were used to remove 15 mg/L Cr(VI). At 15, 30, 45, 60, 120, 240, and 360 min, the impact of contact time on the adsorption efficiency (R%) of the hydrogels was investigated. These hydrogels showed varying abilities to capture Cr(VI), as illustrated in Figure 2a. H3 removed Cr(VI) the fastest at first (R = 92.89% after 15 min) due to the presence of more free nitrogen and oxygen functional groups from its high content of N–CQDs, but then the removal rate slowed down. H1 had the highest R% (99.41%). Despite exposure times of 45, 240, 120, and 120 min for samples HB, H1, H2, and H3, respectively, there was no substantial rise in the adsorption rate for any of the samples. After 45, 240, 120, and 120 min for HB, H1, H2, and H3, respectively, the adsorption rate decreased from 99.28, 99.41, 98.90, and 97.41% to 88.37, 88.37, 96.77, and 88.37%, respectively. This may be due to the leaching process [17]. The hydrogel with the lowest content of N–CQDs (i.e., H1) showed the best adsorption efficiency for Cr(VI) (99.41%). This may be due to the presence of more free functional groups compared to H2 and H3, which are blocked by the high content of N–CQDs.

This study compares the R% of Cr(VI) by the H1 hydrogel with that of hydrogels reported as adsorbents in previous studies. Vilela et al. demonstrated that the maximum R% by chitosan-based hydrogel was 94.72% [22]. In addition, the q_exp._ presented values were between 12 and 13 mg/g for gelatin-based hydrogels compared to our study (i.e., 15.39 mg/g) [23]. The performance of the prepared H1 adsorbent for removing Cr(VI) is similar to that of other low-cost adsorbents. 

Zero-order reaction kinetics are not appropriate for modeling the adsorption of Cr(VI) onto HB, H1, H2, and H3 because they assume that the reaction rate is independent of the concentration of the reactants (Figure 2b) [2]. Since the zero-order reaction model is not appropriate for modeling the adsorption kinetics of Cr(VI) on HB, H1, H2, and H3, pseudo-first-order and pseudo-second-order models were used instead (Figure 2c,d). According to the q_exp._ and R^2^ values in Table 1, the pseudo-first-order model better fits the adsorption data for HB (q_exp.1_ = 15.30 mg/g, R^2^ = 0.9790) and H3 (q_exp.1_ = 15.08 mg/g, R^2^ = 0.9711). This suggests that chemical bonds are involved in the adsorption process. 

In contrast, the R^2^ of H1 and H2 (i.e., 0.930 and 0.899) better fit the pseudo-second-order model. In spite of being obtained using a pseudo-second-order model, the values are still appropriate for characterizing the kinetics of Cr(VI) adsorption onto H1 and H2 (i.e., 15.39 and 15.31 mg/g) by the pseudo-first order. So, the data presented reveal the adsorption mechanisms occurring on the surface, including both chemisorption and physisorption, the adsorption of Cr(VI) by H1 and H2 [2].

The intraparticle diffusion plots in Figure 2e show that the lines do not pass through the origin, indicating two stages of adsorption: surface adsorption and intraparticle diffusion. This means that surface adsorption controls the overall adsorption of Cr(VI) onto HB, H1, H2, and H3. This is attributed to the strong electrostatic attraction between Cr(VI) and the surfaces of HB, H1, H2, and H3, followed by the diffusion of Cr(VI) into their pores [2,24]. According to the Boyd model, external diffusion was not the only rate-controlling step, because the linear modelling did not cross the axis at the origin (Figure 2f). As a result, the Boyd model’s lowest slope is largely linked to a substantial intraparticle diffusion effect. Additionally, the Boyd model’s R^2^ provided a superior fit to the adsorption data of HB, H1, H2, and H3 (i.e., external diffusion). The surface processes, including both external and intraparticle diffusion in the HB, H1, H2, and H3 adsorption of Cr(VI), are clarified by these data. The highest value of α for H3 (i.e., 1.36 mg/g/min.), because of the greatest surface area, are due to the high content of N–CQDs (Figure 2g) [2,20]. 

### 2.2. Chromium Reduction Study

**Figure 3:**
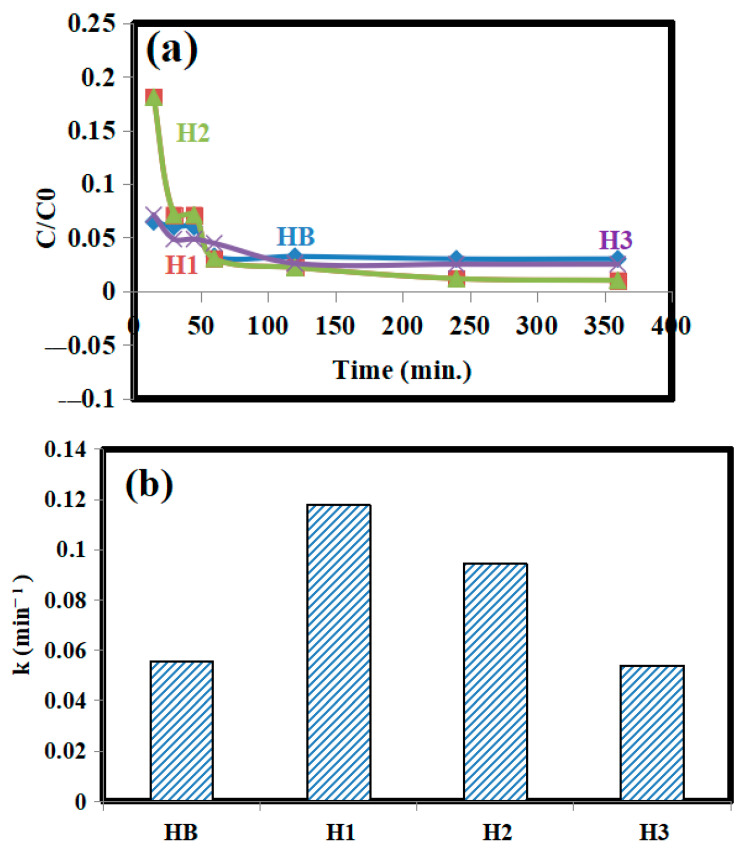
(**a**) Reduction of Cr(VI) by using HB, H1, H2, and H3; (**b**) the obtained reaction rate constants k.

The analysis in Figure 3 suggests that material H1 reduced Cr(VI) to Cr(III) faster than the other materials (HB, H2, and H3). This could be due to the presence of N–CQDs in H1, which are absent in HB, and because H1 has more free functional groups compared to H2 and H3, where these groups are blocked by the high content of N–CQDs. From this we can say that the released Cr(VI), which are de-adsorbed from the prepared hydrogels after a definite time because of the leaching process, could be reduced to Cr(III) to become nontoxic.

### 2.3. HEC-N–CQDs-g-poly(AMPS)-Based Fluorescent Chemosensor for Sensitive Detection of Cr(VI)

Cr(VI) is a serious threat to the environment and human health, so it is important to develop sensitive methods for detecting it. Our previously synthesized HEC-N–CQDs-g-poly(AMPS) hydrogels meet the requirements of a fluorescent probe, so we explored their use for Cr(VI) detection. H1 had the highest R% and the fastest reduction effect for Cr(VI) compared to HB, H2, and H3. As a result, H1 was chosen as the more suitable probe for further studies. 

#### 2.3.1. UV Spectra 

**Figure 4:**
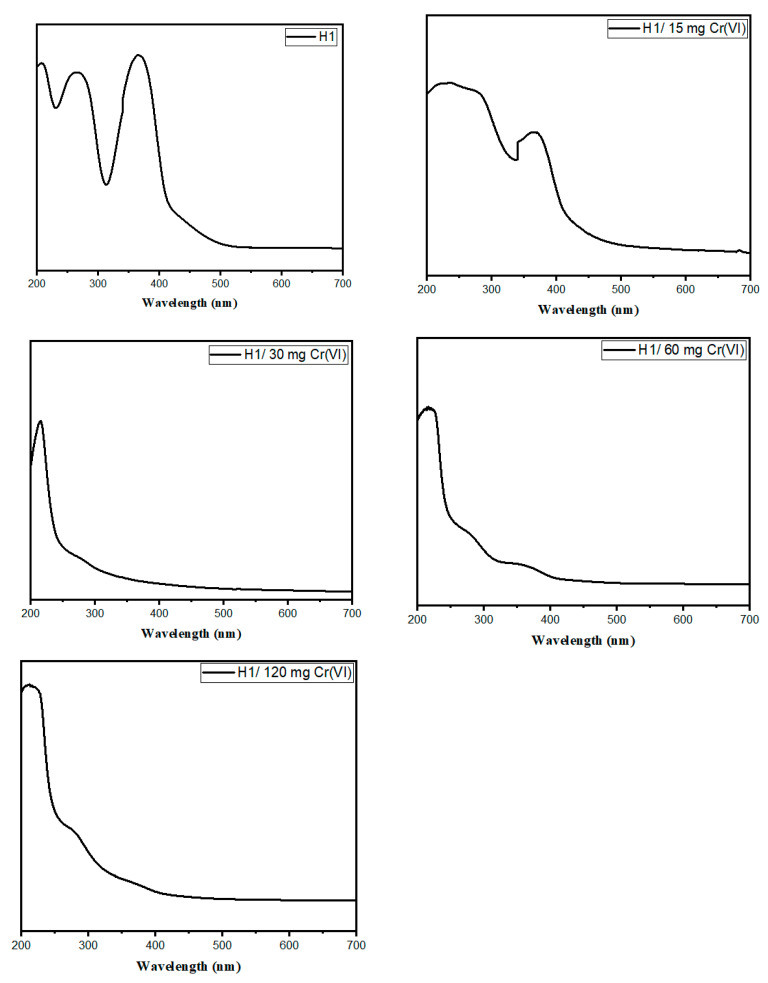
UV spectra for H1 before Cr(VI) adsorption, H1/15 mg Cr(VI), H1/30 mg Cr(VI), H1/60 mg Cr(VI), and H1/120 mg Cr(VI).

The UV–vis spectrum of H1 before Cr(VI) adsorption and H1 after adsorption with different Cr(VI) concentrations(i.e. H1/15 mg Cr(VI), H1/30 mg Cr(VI), H1/60 mg Cr(VI) and H1/120 mg Cr(VI)) in Figure 4 depicts a characteristic absorption of light in the UV region, with the absorption effect diminishing gradually (tailing off) into the visible range of light. 

The spectrum has three main absorption peaks: an intensive first peak at 207, 213, 217, 219, and 220 nm due to the π–π* transition of C=C and –C–C– bonds present in the sp2 carbon electrons in the core structure of the N–CQDs at H1/15 mg Cr(VI), H1/30 mg Cr(VI), H1/60 mg Cr(VI) and H1/120 mg Cr(VI), respectively [2,25]. A second shoulder peak at 276, 268, 265, 264, and 262 nm was observed in the n– π* transition of C=O bonds for H1 before Cr(VI) adsorption, H1/15 mg Cr(VI), H1/30 mg Cr(VI), H1/60 mg Cr(VI) and H1/120 mg Cr(VI), respectively. In addition, a third peak at 373, 370, 280, 363, and 362 nm was assigned to the n–π* transition of S=O bonds for H1 before Cr(VI) adsorption, H1/15 mg Cr(VI), H1/30 mg Cr(VI), H1/60 mg Cr(VI), and H1/120 mg Cr(VI), respectively. The decrease in the wavelength number and the intensity of C=O and S=O peaks for H1/120 mg Cr(VI) was due to the chemical reaction of C=O and S=O with Cr(VI) [2]. This is attributed to the complexation between Cr(VI) and H1 hydrogel. Complex formation is made through the complexation of a polymeric donating ligand (–COO^−^ and SO_3_H) with Cr(VI) [26]. The order in which Cr(VI) forms increasingly stable complex species is H1/120 mg Cr(VI) > H1/60 mg Cr(VI) > H1/30 mg Cr(VI) > H1/15 mg Cr(VI) > H1 without Cr(VI), confirming the trend of chelating stability for Cr(VI). The calculated QY for H1 before Cr(VI) adsorption, H1/15 mg, H1/30 mg, H1/60 mg, and H1/120 mg were 0.051, 0.053, 1.513, 2.057, and 13.718%, respectively. This is will also be proved by the fluorescence images below, which show the high F.I. of H1/120 mg.

#### 2.3.2. Fluorescence Spectroscopy and Microscopy

**Figure 5:**
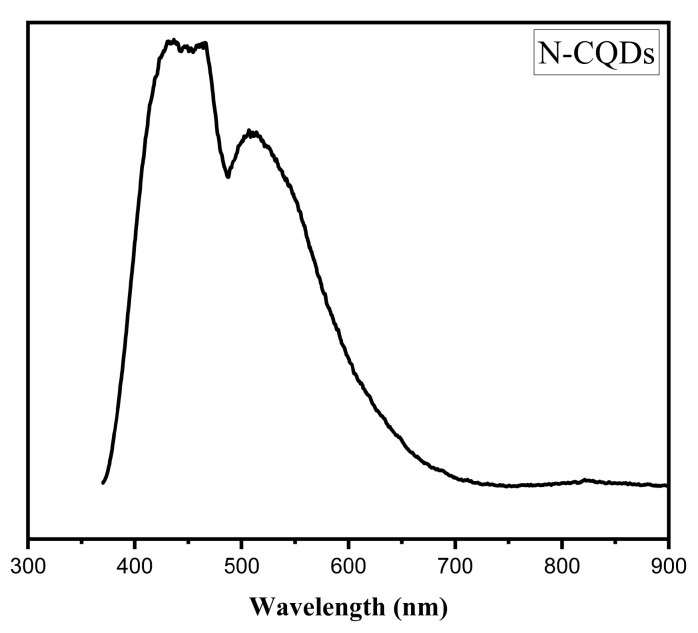
Fluorescence spectra for N–CQDs.

When N–CQDs were excited at 350 nm, their fluorescence emission spectra (Figure 5) exhibited two peaks. The strongest emission response was at a wavelength (λ) of 449 nm, attributed to C=O/C=N moieties on the N–CQDs’ surfaces. An additional peak at 514 nm is ascribed to oxygen vacancy defects [2,8]. The N–CQDs’ calculated QY was 16.50%.

Based on the previously researched fluorescent properties of HEC-N–CQDs-g-poly(AMPS) hydrogels, different concentrations of Cr(VI) were added to the aqueous solution of H1, and the fluorescence intensity (F.I.) was measured in order to study the sensitivity of the H1 chemosensor. After the addition of Cr(VI), the Cr(VI) adsorbed on the H1 surface and generated an electrostatic repulsion with the positive ions of N–CQDs. By improving the dispersion of N–CQDs in H1, the negative effects of aggregation-caused quenching (ACQ) were able to be weakened. As a result, Cr(VI) adsorbed onto H1 can enhance F.I. [27].

**Figure 6:**
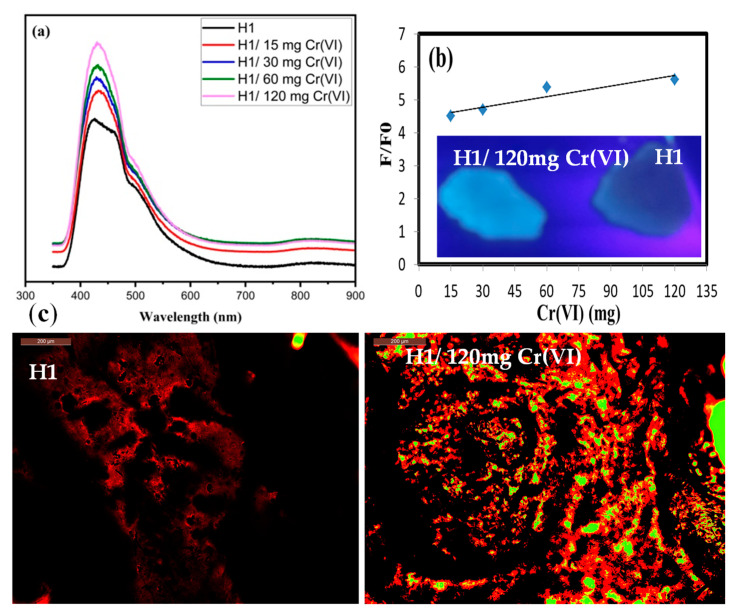
(**a**) Fluorescence spectra for H1 before Cr(VI) adsorption, H1/15 mg Cr(VI), H1/30 mg Cr(VI), H1/60 mg Cr(VI), and H1/120 mg Cr(VI); (**b**) plot for relative fluorescence intensity in the presence of various concentrations of Cr(VI) with images by camera under UV light (the UV images are represented as inset); (**c**) fluorescence microscope images of H1 before adsorption and H1/120 mg Cr(VI).

The H1 adsorbed Cr(VI) with different concentrations, i.e., 0, 15, 30, 60, and 120 mg/L, were excited at 350 nm and emitted the strongest response λ at 430, 433, 433, 433, and 435 nm, respectively. As shown in Figure 6a, the F.I. of the H1 at around 430 nm increases gradually when the Cr(VI) concentration increases, indicating that the addition of Cr(VI) can effectively increase the fluorescence of the H1. Figure 6a shows that the fluorescence of H1 is the highest after adsorbing 120 mg Cr(VI). This is due to the high content of N–CQDs, which weaken ACQ effects. The intermolecular π-π/n-π* interactions or other non-radiative channels prevent H1 from releasing its full potential of light emission compared to H1/120 mg Cr(VI) [28,29]. This is also proved by the images taken by camera under UV light, which showed a fluorescence enhancement after the adsorption of Cr(VI) by H1 (Figure 6b). Hence, 120 mg/L was used as the suitable concentration in further applications. 

So, we can conclude that the chromium-sensing ability of the H1 likely arises from preventing the clumping of N–CQDs because of Cr(VI) adsorption, which reduces a phenomenon called ACQ and allows the N–CQDs to fluoresce more brightly [2]. While the intrinsic properties of the N–CQDs play a crucial role, these interactions with Cr(VI) act as amplifiers, leading to the observed increase in fluorescence.

Figure 6b shows a positive correlation between the F.I. of H1 and the concentration of Cr(VI). As the concentration of Cr(VI) gradually increases from 15 mg/L to 120 mg/L, there is a noticeable improvement in the F.I.’s performance. From this we can say that the HEC-N–CQDs-g-poly(AMPS) hydrogel creates a fluorescence assay system that is sensitive to Cr(VI). Figure 6b indicates a good linear relationship between the relative F.I. and the Cr(VI) concentration from 15 mg/L to 120 mg/L. The LOD was measured to be 0.0053 mg/L, which is lower than the WHO guideline value of 50 µg/l for drinking water [29]. 

When observing H1 and H1/120 mg Cr(VI) under a fluorescence microscope, a strong red fluorescence was only seen in the H1/120 mg Cr(VI), which is attributed to the ACQ effect discussed earlier (Figure 6c). 

### 2.4. Morphological Observations

**Figure 7:**
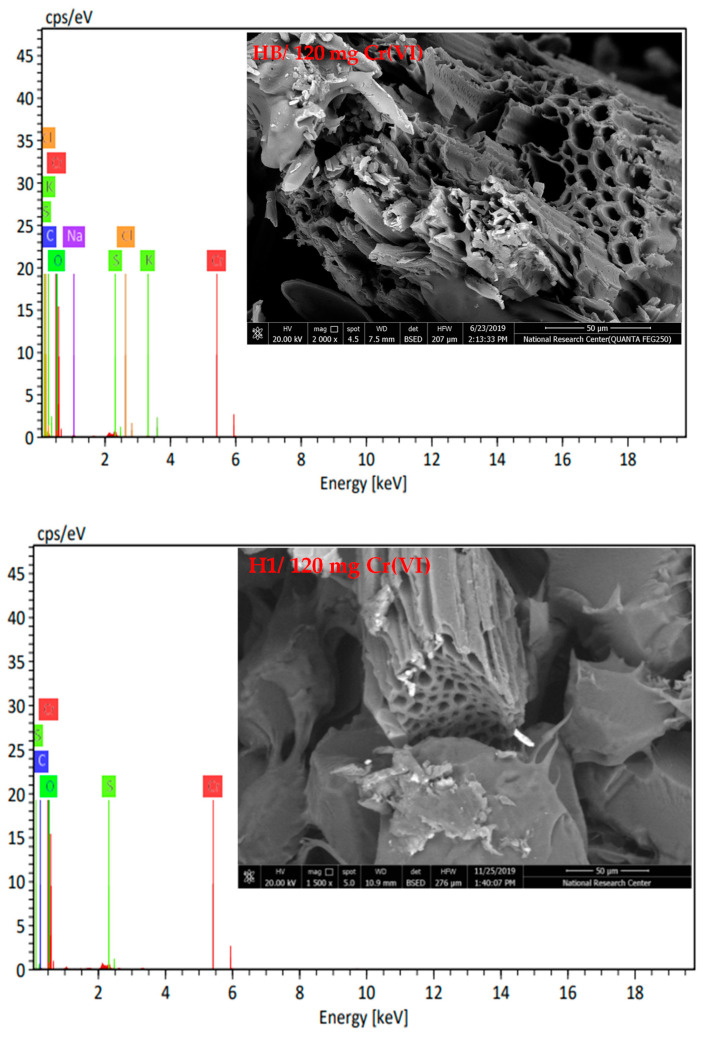
SEM/EDS analysis of HB/120 mg Cr(VI) and H1/120 mg Cr(VI).

Figure 7 depicts the surface morphology of the HB/120 mg Cr(VI) and H1/120 mg Cr(VI) hydrogels, as well as the Cr(VI) percentage. It was discovered that the Cr(VI) percentage in EDX was 0.78 and 1.16%.

### 2.5. Fourier Transform Infrared Spectroscopy (FTIR) Spectra

**Figure 8:**
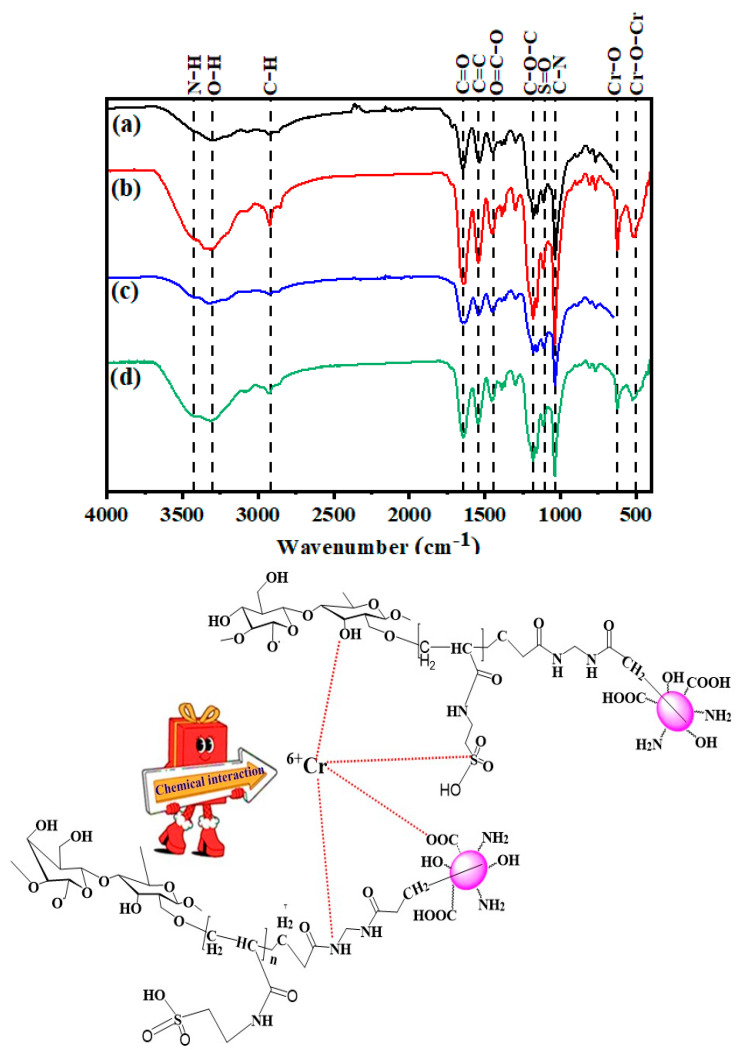
FTIR spectra of (a) HB before adsorption, (b) HB/120 mg Cr(VI), (c) H1 before adsorption, and (d) H1/120 mg Cr(VI) (**upper**), and the mechanism of Cr(VI) adsorption on HEC–N–CQDs–g-poly(AMPS) hydrogels (**lower**).

Figure 7 shows the FTIR spectra of HB and H1 before and after Cr(VI) adsorption. The HB, HB/120 mg Cr(VI), H1, and H1/120 mg Cr(VI) showed absorption bands between 3411–3427 (N–H), 3303–3326 (O–H), 2921–2927 (C–H), 1629–1641 (C=O), 1538–1544 (C=C), 1444–1452 (O=C–O), 1172–1180 (C–O–C), 1110–1114 (S=O), and 1035–1037 cm^−1^ (C–N) [8]. For HB/120 mg Cr(VI) and H1/120 mg Cr(VI), additional new bands appeared between 622–624 (Cr–O) and 520–522 (Cr–O–Cr) cm^−1^ due to Cr(VI) adsorption (Figure 8) [30].

One can observe an increase in the LOI value upon Cr(VI) adsorption onto HB and H1, from 0.33 (HB), 0.34 (H1) to 1.60 (HB/120 mg Cr(VI)), and 1.29 (H1/120 mg Cr(VI)). Upon Cr(VI) adsorption, the LOI increased because of the bonding between Cr(VI) and the hydrogels by chemical interaction. This can be proved by the shifting of the OH and C=O groups positions after Cr(VI) adsorption. Slight shifting was observed on the N–H band (from 3415 to 3421 cm^−1^ for HB/120 mg Cr(VI) and from 3421 to 3425 cm^−1^ for H1/120 mg Cr(VI)) and on the C=O band (from 1645 to 1633 cm^−1^ for HB/120 mg Cr(VI) and from 1646 to 1637 cm^−1^ for H1/120 mg Cr(VI)). The band for OH was shifted from 3326 to 3307 cm^−1^ for HB/120 mg Cr(VI) and 3315 to 3303 cm^−1^ for H1/120 mg Cr(VI), which means that N–H, C=O, and OH are involved in the binding of Cr(VI) [2,8]. The Cr(VI) form complexes with the HB and H1, such as through ion exchange reactions, as shown in Figure 8. A lower OH band wave number after Cr(VI) adsorption proves the stronger intermolecular H-bonding between HB and H1, and Cr(VI). In addition, the N–H, C=O, and S=O groups could bond with Cr(VI), as a result of shifting the N–H, C=O, and S=O stretching frequencies to lower wave numbers, as shown in Table 2 [8]. The FTIR spectrum of the HB/120 mg Cr(VI) and H1/120 mg Cr(VI) differed in their Cr(VI) adsorption capability, proved by the increased relative absorbance (RA) of the absorbance bands of Cr–O (i.e., 0.77 for HB/120 mg Cr(VI) and 0.87 for H1/120 mg Cr(VI)) and Cr–O–Cr (i.e., 0.81 for HB/120 mg Cr(VI) and 0.89 for H1/120 mg Cr(VI)). The increased RA for the Cr–O and Cr–O–Cr bands for H1/120 mg Cr(VI) compared to HB/120 mg Cr(VI) improved its R%, which was proved in the adsorption part above.

## 3. Conclusions

The microwave method was used to prepare N–CQDs from MB. This method is advantageous due to its low cost and ease of use. The prepared N–CQDs were used to create the HEC-N–CQDs-g-poly(co-AMPS) hydrogels. H1’s high adsorption (99.41%) and reduction rate likely result from N–CQDs (absent in HB) and more free functional groups (vs. H2/H3, where N–CQDs block them). This promotes Cr(VI) desorption from the hydrogels, increasing its availability for reduction to Cr(III). For these reasons, H1 was selected as the more appropriate probe for additional research. Different concentrations of Cr(VI) were added to the aqueous solution of H1, and the F.I. was measured in order to study the sensitivity of the H1 chemosensor. It was shown that the F.I. increase with high concentration. This is attributed to the high electrostatic repulsion between Cr(VI) and N–CQDs, which weaken the ACQ effect. The fluorescence microscope showed that H1/120 mg Cr(VI) has higher fluorescence intensity compared to H1. The calculated LOD was 0.0053 mg/L, which is less than the WHO recommended drinking water detection level. 

The FTIR spectroscopy proved the complexation between Cr(VI) and H1. Additional new bands appeared between 622–624 (Cr–O) and 520–522 (Cr–O–Cr) cm^−1^ due to Cr(VI) adsorption. It was also proved that N–H, OH, C=O and S=O groups enter in the complexation. From our results, we can say that HEC-N–CQDs-g-poly(co-AMPS) hydrogels could be used as effective adsorbents and chemosensors.

## 4. Materials and Methods

### 4.1. Materials

MB waste from a carpentry workshop was air-dried. AMPS (2-Acrylamido-2-methyl-1-propane-sulfonic acid), MBA (N,N′-methylenebisacrylamide) and KPS (potassium persulfate) were purchased from Sigma-Aldrich (Saint Louis, MO, USA). In this investigation, analytical-grade chemicals, reagents, and substrates were all utilized exactly as supplied, requiring no additional purification. 

### 4.2. Preparation of HEC-N–CQDs-g-poly(co-AMPS) 

We synthesized HEC-N–CQDs-g-poly(co-AMPS) hydrogels in our previous published work by graft copolymerizing and cross-linking AMPS and N–CQDs into HEC. We coded these hydrogels as H1 (contain 5% N–CQDs), H2 (contain 10% N–CQDs), and H3 (contain 15% N–CQDs). We also prepared a blank hydrogel without N–CQDs, which we labeled as HB (Table 3) [8].

### 4.3. Characterization and Analysis

#### 4.3.1. Chromium Adsorption Study

We compared the removal efficiency (R%) and adsorption capacity (qe) of HB, H1, H2, and H3 by using 20 mg of each hydrogel in 20 mL of Cr(VI) solution at a concentration of 15 mg/L, a pH of approximately 5, and different contact times (i.e., 15, 30, 45, 60, 120, 240, and 360 min). We used Equations (1) and (2) to evaluate the adsorption efficiencies of HB, H1, H2, and H3.

(1) $$R\%=\frac{\left(C0-Ct\right)}{C0}100$$

(2) $$qe=\frac{\left(C0-Ct\right)}{m}V$$

where Co and Ct represent the starting and ending concentrations of Cr(VI) in solution (mg/L); V is the volume of solution (l); and m is the mass of the hydrogel (g) used to remove Cr(VI) [2].

Zero-order, pseudo-first-order, and pseudo-second-order kinetic models can be estimated by Equations (3)–(5).

C_e_ = C_o_ − K_0_ (3)

ln [q_e1_ − q_t_] = ln q_e1_ − K_1_t (4)

(5) $$\frac{t}{qt}=\frac{1}{K2qe2}-\frac{t}{qt}$$

where qe and qt represent the amounts of Cr(VI) adsorbed (mg/g) at equilibrium sorption capacity and time (t), respectively; Ce is the ending concentration of Cr(VI) with contact time (t); K_1_ (min^−1^) is the pseudo-first-order rate constant of adsorption; and K_2_ is the rate constant of pseudo-second-order adsorption. The values of q_e2_ and K_2_ can be calculated from the slope and intercept of the plot of t/qt versus t, respectively [2,18].

The Weber–Morris intraparticle diffusion can be estimated using Equation (6):

qt = K_3_ t^1/2^ + C_3_ (6)

where K_3_ is the intraparticle diffusion rate constant, and C_3_ is the slope representing the thickness of the boundary layer [2]. 

The Boyd and Elovich models can be estimated using Equations (7) and (8).

B_t_ = –0.4977 − ln(1 − F) (7)

(8) $$qt=\frac{\ln\alpha b}{b}\;-\frac{1}{b}\;\ln t$$

where Bt is a mathematical function of F, similar to qt/qe, that reflects the amount of HB or H1 adsorbed at different periods. The initial rate of adsorption (α) is measured in mg/g/min, while b represents the extent of surface coverage and activation energy for chemisorption (g/mg). Plotting qt against ln t yields a straight line, with α and b determined from the slope (1/b) and intercept (ln αb/b), respectively. The Elovich model can be used to study the adsorption rate on heterogeneous surfaces based on their absorption capacity. This model is also used to represent pseudo-second-order kinetics (i.e., chemisorption), when the sorbent surface has a heterogeneous energy distribution [2]. 

#### 4.3.2. Chromium Reduction Study

Researchers used the reaction rate constant (k) to compare how effectively hydrogels promote the reduction of Cr(VI), which is defined as follows:

(9) $$k=\ln\;\frac{C0}{C}/t$$

The equation describes the change in concentration of Cr(VI) over time. The initial concentration is represented by C₀, and C represents the concentration at a specific time (t) [31]. 

#### 4.3.3. Fourier-Transform Infrared (FTIR) Spectra 

FTIR were taken with a Mattson 5000 spectrometer (Unicam, Bedfordshire, UK) using the potassium bromide (KBr) disk method. The crystallinity index (LOI) was calculated using the equations below:

(10) $$\mathrm{LOI}=\frac{A_{OH}}{A_{CH}}$$

where A_1425_ and A_900_, A_OH_ and A_CH_ refer to the FTIR absorbance at 1425 cm^−1^, 900 cm^−1^, OH, and CH bands [8,18]. 

#### 4.3.4. SEM/EDX

Images were captured using a Quanta/250-FEG scanning electron microscope (Thermo Fisher Scientific, Waltham, MA, USA) equipped with an energy-dispersive X-ray analyzer. The acceleration voltage was 30 kV.

#### 4.3.5. Fluorescence Microscope

Fluorescence microscopy was performed using a Jasco FP-6500 spectrofluorometer (Tokyo, Japan) with a 150-watt xenon arc lamp. It is sourced from NRC, Cairo, Egypt.

#### 4.3.6. Fluorescence Spectra

The limit of detection (LOD) was detected according to the following equation:

(11) $$\mathrm{LOD}=\frac{3\sigma }{Slope}$$

where σ refers to the standard deviation of the response at different concentrations of Cr(VI) and slope refers to the slope of the calibration curve [2].

#### 4.3.7. UV Spectra

A UV-Vis spectrophotometer (JASCO V-630, Tokyo, Japan) was used to record the UV–vis absorption spectra on a quartz cell with a path length of 1 cm. The quantum yield (QY) was estimated as follows:

(12) $$\;\;QY=Q_{st.}\frac{m_{X}}{m_{st.}}(\frac{{ɳ}_{X}}{{ɳ}_{st.}}{)}^{2}$$

where “QY” is the quantum yield, “m_x_” is the slope from the fluorescence versus absorbance plot, “ɳ” is the refractive index of the solvent, and “m_st._” refers to the methylene blue standard solution in water (0.1 M).

## Figures and Tables

**Table 1:** This study compares the effectiveness of different models in describing the adsorption process for HB, H1, H2, and H3.

Parameter	HB	H1	H2	H3
**K_0_**	81 × 10^−3^	46 × 10^−2^	83 × 10^−2^	42 × 10^−3^
**R^2^**	0.713	0.814	0.800	0.815
**q_exp_. (mg/g)**	15.30	15.39	15.31	15.08
**q_Calc.1_ (mg/g)**	15.67	12.79	10.87	15.00
**K_1_**	56 × 10^−4^	56 × 10^−4^	61 × 10^−3^	37 × 10^−4^
**R^2^**	0.979	0.835	0.800	0.971
**q_Calc.2_ (mg/g)**	3.48	3.56	2.15	2.18
**K_2_**	70 × 10^−3^	45 × 10^−2^	65 × 10^−1^	21 × 10^−2^
**R^2^**	0.881	0.930	0.899	0.916
**C_3_**	0.19	0.41	0.65	0.11
**K_3_**	0.97	3.91	6.10	0.01
**R^2^**	0.831	0.812	0.830	0.937
**Slope_Boyd_**	45 × 10^−4^	18 × 10^−4^	62 × 10^−4^	31 × 10^−4^
**Intercept_Boyd_**	14 × 10^−2^	16 × 10^−1^	12 × 10^−1^	40 × 10^−2^
**R^2^**	0.977	0.814	0.892	0.983
**α (mg/g/min.)**	1.03	0.80	0.91	1.36
**b (g/mg)**	1.04	1.35	1.10	1.70
**R^2^**	0.908	0.949	0.811	0.908

**Table 2:** MHBS and LOI of HB before adsorption, HB/120 mg Cr(VI), H1 before adsorption, and H1/120 mg Cr(VI).

Sample	$\mathrm{LOI}\;(\frac{A_{1425}}{A_{900}})$	N–H Position (cm^−1^)	OH Position (cm^−1^)	C=O Position (cm^−1^)	S=O Position (cm^−1^)
**HB**	0.33	3326	3295	1645	1114
**HB/120 mg Cr(VI)**	1.60	3307	3307	1633	1112
**H1**	0.34	3315	3290	1646	1112
**H1/120 mg Cr(VI)**	1.29	3303	3303	1637	1110

**Table 3:** The composition and percentage of the prepared HEC-N–CQDs-g-poly(co-AMPS) hydrogels.

Hydrogel Code	N–CQDs (%)	HEC (gm)	KPS (gm)	AMPS (gm)	MBA (gm)
**H0**	0	2	0.24	4	0.48
**H1**	5	2	0.24	4	0.48
**H2**	10	2	0.24	4	0.48
**H3**	15	2	0.24	4	0.48

## Data Availability

The original contributions presented in the study are included in the article material, further inquiries can be directed to the corresponding author.

## References

[1] Wang D.-C., Lei S.-N., Zhong S., Xiao X., Guo Q.-H. (2023). Cellulose-Based Conductive Materials for Energy and Sensing Applications. Polymers.

[2] Tohamy H.-A.S., El-Sakhawy M., Kamel S. (2023). Microwave-assisted synthesis of amphoteric fluorescence carbon quantum dots and their chromium adsorption from aqueous solution. Sci. Rep..

[3] Kumar V., Dwivedi S.K. (2021). A review on accessible techniques for removal of hexavalent Chromium and divalent Nickel from industrial wastewater: Recent research and future outlook. J. Clean. Prod..

[4] Jobby R., Jha P., Yadav A.K., Desai N. (2018). Biosorption and biotransformation of hexavalent chromium [Cr (VI)]: A comprehensive review. Chemosphere.

[5] Tohamy H.A.S., El-Sakhawy M., Elnasharty M.M. (2023). Carboxymethyl cellulose membranes blended with carbon nanotubes/ag nanoparticles for eco-friendly safer lithium-ion batteries. Diam. Relat. Mater..

[6] Yang W.-M., Liu F., Jin Y.-T., Dong Z.-M., Zhao G.-C. (2022). Efficient reduction of Cr (VI) with carbon quantum dots. ACS Omega.

[7] Qian Z., Ma J., Shan X., Feng H., Shao L., Chen J. (2014). Highly luminescent N-doped carbon quantum dots as an effective multifunctional fluorescence sensing platform. Chem. A Eur. J..

[8] Tohamy H.-A.S. (2023). Cellulosic nitrogen doped carbon quantum dots hydrogels with fluorescence/visco-elastic properties for pH-and temperature-sensitivity. Diam. Relat. Mater..

[9] Cao M., Zhao X., Gong X. (2022). Ionic liquid-assisted fast synthesis of carbon dots with strong fluorescence and their tunable multicolor emission. Small.

[10] Li J., Zheng S., Zhang S., Gong X. (2024). Metal–Organic Framework-Assisted Rational Design of Multicolor Solid-State Fluorescent Carbon Nanodots and Its Application for LEDs. Laser Photonics Rev..

[11] Shen C.L., Lou Q., Lv C.F., Zang J.H., Qu S.N., Dong L., Shan C.X. (2019). Bright and multicolor chemiluminescent carbon nanodots for advanced information encryption. Adv. Sci..

[12] Meng X., Song Y., Jing Q., Zhao H. (2023). Self-precipitation of highly purified red emitting carbon dots as red phosphors. J. Phys. Chem. Lett..

[13] Nguyen K.G., Baragau I.-A., Gromicova R., Nicolaev A., Thomson S.A., Rennie A., Power N.P., Sajjad M.T., Kellici S. (2022). Investigating the effect of N-doping on carbon quantum dots structure, optical properties and metal ion screening. Sci. Rep..

[14] Pang B., Jiang G., Zhou J., Zhu Y., Cheng W., Zhao D., Wang K., Xu G., Yu H. (2021). Molecular-scale design of cellulose-based functional materials for flexible electronic devices. Adv. Electron. Mater..

[15] Mitchell S., Qin R., Zheng N., Pérez-Ramírez J. (2021). Nanoscale engineering of catalytic materials for sustainable technologies. Nat. Nanotechnol..

[16] Varma R.S. (2019). Biomass-derived renewable carbonaceous materials for sustainable chemical and environmental applications. ACS Sustain. Chem. Eng..

[17] Stern R., Jedrzejas M.J. (2008). Carbohydrate polymers at the center of life’s origins: The importance of molecular processivity. Chem. Rev..

[18] Tohamy H.A.S. (2024). Oil dispersing and adsorption by carboxymethyl cellulose–oxalate nanofibrils/nanocrystals and their kinetics. J. Surfactants Deterg..

[19] Slavkova M., Tzankov B., Popova T., Voycheva C. (2023). Gel formulations for topical treatment of skin cancer: A review. Gels.

[20] Bao Y., Ma J., Li N. (2011). Synthesis and swelling behaviors of sodium carboxymethyl cellulose-g-poly (AA-co-AM-co-AMPS)/MMT superabsorbent hydrogel. Carbohydr. Polym..

[21] Ma J., Li X., Bao Y. (2015). Advances in cellulose-based superabsorbent hydrogels. RSC Adv..

[22] Vilela P.B., Dalalibera A., Duminelli E.C., Becegato V.A., Paulino A.T. (2019). Adsorption and removal of chromium (VI) contained in aqueous solutions using a chitosan-based hydrogel. Environ. Sci. Pollut. Res..

[23] Marciano J.S., Ferreira R.R., de Souza A.G., Barbosa R.F., de Moura Junior A.J., Rosa D.S. (2021). Biodegradable gelatin composite hydrogels filled with cellulose for chromium (VI) adsorption from contaminated water. Int. J. Biol. Macromol..

[24] Raoov M., Mohamad S., Abas M.R. (2013). Synthesis and characterization of β-cyclodextrin functionalized ionic liquid polymer as a macroporous material for the removal of phenols and As (V). Int. J. Mol. Sci..

[25] Torres F.G., Gonzales K.N., Troncoso O.P., Cañedo V.S. (2023). Carbon Quantum Dots Based on Marine Polysaccharides: Types, Synthesis, and Applications. Mar. Drugs.

[26] Soykan C., Coskun R., Kirbag S. (2007). Poly (crotonic acid-co-2-acrylamido-2-methyl-1-propanesulfonic acid)–metal complexes with copper (II), cobalt (II), and nickel (II): Synthesis, characterization and antimicrobial activity. Eur. Polym. J..

[27] Wang Z., Xiao X., Yang Y., Zou T., Xing X., Zhao R., Wang Z., Wang Y. (2019). L-Aspartic acid capped CdS quantum dots as a high performance fluorescence assay for sliver ions (I) detection. Nanomaterials.

[28] Venkatesvaran H., Balu S., Chowdhury A., Chen S.-W., Yang T.C.-K. (2022). Photo–Redox Properties of–SO3H Functionalized Metal-Free g-C3N4 and Its Application in the Photooxidation of Sunset Yellow FCF and Photoreduction of Cr (VI). Catalysts.

[29] Zhu X., Deng Y., Li P., Yuan D., Ma J. (2019). Automated syringe-pump-based flow-batch analysis for spectrophotometric determination of trace hexavalent chromium in water samples. Microchem. J..

[30] Vats V., Melton G., Islam M., Krishnan V.V. (2023). FTIR spectroscopy as a convenient tool for detection and identification of airborne Cr (VI) compounds arising from arc welding fumes. J. Hazard. Mater..

[31] Mushtaq F., Chen X.-z., Veciana A., Hoop M., Nelson B.J., Pané S. (2022). Magnetoelectric reduction of chromium (VI) to chromium (III). Appl. Mater. Today.

